# Poster Session I - A100 IMPACT OF GLP-1 RECEPTOR AGONISTS ON THE QUALITY OF BOWEL PREPARATION IN SCREENING COLONOSCOPIES

**DOI:** 10.1093/jcag/gwaf042.100

**Published:** 2026-02-13

**Authors:** S Quan, E J Cheng

**Affiliations:** University of Calgary Cumming School of Medicine, Calgary, AB, Canada; University of Calgary Cumming School of Medicine, Calgary, AB, Canada

## Abstract

**Background:**

The effectiveness of a colonoscopy is dependent on the quality of the bowel preparation; the Boston Bowel Preparation Scale (BBPS) is a measure used to assess bowel preparation quality. The American Gastroenterology Association recommends a BBPS of ≥ 6 for screening colonoscopies. GLP-1 receptor agonists (GLP-1RA) are a class of medications used for treating a variety of conditions, acting to increase insulin secretion and reduce gastrointestinal motility. The effect on motility has raised questions about the impact these medications have on the quality of bowel preparations.

**Aims:**

The aim of this study was to examine the effect of semaglutide on the quality of bowel preparation for patients undergoing colonoscopies for colorectal cancer screening. We describe the BBPS scores of patients undergoing screening colonoscopies and compare the scores between the two groups. We also compare the proportions of patients who have a BBPS <6 based on their usage of semaglutide and describe the average BBPS of patients in each group.

**Methods:**

This was a retrospective study using administrative data collected from the colorectal cancer screening program at the Foothills Medical Centre from January to December 2024. BBPS scores for patients using and not using semaglutide (based on collected medication histories) were extracted from the program’s electronic health information system (Connect Care).

**Results:**

The BBPS for 18359 patients were extracted and classified based on their use of semaglutide. The majority of patients (95.6% of the study population) had a BBPS ≥6. A Mann Whitney U Test showed there was a statistically significant difference in BBPS between patients using semaglutide and not using semaglutide (U = 6310100, p < 0.001).Of the patients on semaglutide, 7.5% had a BBPS score <6, while 2.8% of patients not on semaglutide had a BBPS <6 (X^2^ (1, N = 18074)=52.4, p < 0.001). The mean BBPS for patients on semaglutide was 7.3, compared to 7.9 for patients not on semaglutide.

**Conclusions:**

A significantly higher proportion of patients on semaglutide had a substandard bowel preparation compared to those not on the medication. This suggests GLP-1RA may have an impact on bowel preparation in screening colonoscopies, with potential negative consequences such as missed lesions, increased procedural risks, and increased healthcare costs.

**Funding Agencies:**

None

